# Epoxy Fatty Acids and Inhibition of the Soluble Epoxide Hydrolase Selectively Modulate GABA Mediated Neurotransmission to Delay Onset of Seizures

**DOI:** 10.1371/journal.pone.0080922

**Published:** 2013-12-11

**Authors:** Bora Inceoglu, Dorota Zolkowska, Hyun Ju Yoo, Karen M. Wagner, Jun Yang, Edward Hackett, Sung Hee Hwang, Kin Sing Stephen Lee, Michael A. Rogawski, Christophe Morisseau, Bruce D. Hammock

**Affiliations:** 1 Department of Entomology and UC Davis Comprehensive Cancer Center, University of California Davis, Sacramento, California, United States of America; 2 Department of Neurology, School of Medicine, University of California Davis, Sacramento, California, United States of America; 3 Metabolomics Core Laboratory, Biomedical Research Center, Asan Institute of Life Sciences, Seoul, Korea; University of Graz, Austria

## Abstract

In the brain, seizures lead to release of large amounts of polyunsaturated fatty acids including arachidonic acid (ARA). ARA is a substrate for three major enzymatic routes of metabolism by cyclooxygenase, lipoxygenase and cytochrome P450 enzymes. These enzymes convert ARA to potent lipid mediators including prostanoids, leukotrienes and epoxyeicosatrienoic acids (EETs). The prostanoids and leukotrienes are largely pro-inflammatory molecules that sensitize neurons whereas EETs are anti-inflammatory and reduce the excitability of neurons. Recent evidence suggests a GABA-related mode of action potentially mediated by neurosteroids. Here we tested this hypothesis using models of chemically induced seizures. The level of EETs in the brain was modulated by inhibiting the soluble epoxide hydrolase (sEH), the major enzyme that metabolizes EETs to inactive molecules, by genetic deletion of sEH and by direct administration of EETs into the brain. All three approaches delayed onset of seizures instigated by GABA antagonists but not seizures through other mechanisms. Inhibition of neurosteroid synthesis by finasteride partially blocked the anticonvulsant effects of sEH inhibitors while the efficacy of an inactive dose of neurosteroid allopregnanolone was enhanced by sEH inhibition. Consistent with earlier findings, levels of prostanoids in the brain were elevated. In contrast, levels of bioactive EpFAs were decreased following seizures. Overall these results demonstrate that EETs are natural molecules which suppress the tonic component of seizure related excitability through modulating the GABA activity and that exploration of the EET mediated signaling in the brain could yield alternative approaches to treat convulsive disorders.

## Introduction

Convulsive disorders affect a significant number of people, with a rate of prevalence of 1% in the general population, despite the availability of more than 20 FDA approved anti-convulsive drugs [1]. Increased rate of mortality and reduced quality of life in people suffering from a broad range of epileptic disorders justify the need for improved anti-epileptic drugs [2]. Identification of biological pathways that could be exploited to reduce the excitability of neurons should result in the discovery of more effective and safe therapeutics. Here we report an approach to reduce seizure related excitability by augmentation of the levels of natural epoxy fatty acids (EpFAs) in the brain and a selective mechanism of action for EpFAs on GABA-related signaling.

The natural monoepoxide metabolites of major polyunsaturated fatty acids are bioactive molecules involved in the regulation of neuronal excitability, in particular during pathological processes [3]. Monoepoxides of linoleic, arachidonic (ARA), eicosapentaenoic (EPA) and docosahexaenoic (DHA) acids are naturally produced in the central and peripheral nervous system by a number of cytochrome P450 isozymes [4]. These cytochrome P450s at the same time, constitute the third and least studied branch of the ARA cascade [5]. The prostanoids and leukotrienes are proinflammatory products of the cyclooxygenase and lipoxygenase branches of the cascade and are mostly sensitizing or directly excitatory on neurons. However, the cytochrome P450 produced EpFAs are largely anti-inflammatory and presumably reduce excitation of neurons, specifically of nociceptors [4], [6]. The ARA derived EETs also influence the cross-talk between the three branches of the ARA cascade by suppressing the transcription of pro-inflammatory enzymes in the two other branches [7], [8]. Thus, the EETs and other EpFAs display anti-inflammatory and anti-hyperalgesic effects in models of inflammatory pain [9].

The intracellular concentration of the free EpFAs are tightly regulated by the enzyme soluble epoxide hydrolase (sEH) which converts EpFAs to their corresponding diols, molecules that are inactive or in some cases seem to oppose the activities of EpFAs [10]. Inhibition of the sEH using potent and bioavailable small molecule sEH inhibitors (sEHI) *in vivo* stabilize EpFAs and allow the quantification of their effects on physiologic processes, since in the presence of sEH activity the half life of EpFAs are in the order of seconds [9], [11]. The use of sEHI enabled our group to begin investigating the roles of EpFAs in the nervous system. Subsequently, we reported anti-hyperalgesic and analgesic effects of the EpFAs [3], [9], [12]. However, the spectrum of activity of EpFAs and inhibitors of sEH on nociception well surpass the spectrum of activity of non steroidal anti-inflammatory drugs and selective cox-2 inhibitors as demonstrated in a number of models of pain [12]. Their breadth of activity is inconsistent with the idea that EpFAs are strictly anti-inflammatory molecules. The wider range of analgesic effects of sEH inhibitors, in particular on neuropathic models suggests the presence of additional models of action other than blocking inflammation [13]. Current evidence converges on two potential mechanisms that are independent of inflammation, one involving the endogenous opioid system and the other involving the GABA-ergic system [12], [14].

Possible effects of EpFAs on GABA-ergic transmission may be through neurosteroids [3]. Blockade of steroid synthesis by aminoglutethimide and finasteride impedes, while antagonizing steroid receptors has no effect on anti-hyperalgesia driven by sEH inhibition [3]. The GABA antagonist picrotoxin is an antagonist of sEHI mediated analgesia [12]. We reasoned if EpFAs and sEHI contribute to GABA-ergic signaling they should display anticonvulsant effects. This hypothesis was tested in C57/BL6 sEH knockout as well as Swiss mice using a battery of well characterized assays of chemically and electrically induced seizures. Here we report that natural EpFAs may have a role in setting the GABA-ergic tone and drastic decreases in their levels may be detrimental.

## Materials and Methods

### Ethics Statement

All studies were conducted in line with federal regulations and were approved by the Institutional Animal Care and Use Committee at University of California, Davis.

### Chemicals and synthesis

Synthesis of sEH inhibitors TUPS (1-(1-methylsulfonylpiperidin-4-yl)-3-(4-trifluoromethoxy-phenyl)-urea) and TPPU (1-trifluoromethoxyphenyl-3-(1-propionylpiperidin-4-yl) urea) were previously reported [15], [16]. Synthesis characterization and potency determination of inhibitors of sEH using homogenous, recombinant, affinity purified mouse sEH were performed in our laboratory as described [17]. Methyl esters of unsaturated lipids were purchased from NuChek Prep (Elysian, MN). The epoxides of arachidonic, eicosapentaenoic and docosahexaenoic acids (EETs, EpETEs and EpDPEs respectively) were synthesized by reacting the methyl ester forms of these fatty acids with *meta*-chloroperbenzoic acid (*m*CPBA) and purified by reverse phase HPLC as described previously [18], [19]. Characterization and purity determination were accomplished using LC-MS/MS with full scan in the range of 50–450 m/z which indicated high purity for each of the regioisomer mixtures [20]. These oxylipids were stored under nitrogen at −80°C until use.

The 11(12)-EET*d8 and PGD2*d4 were purchased from Cayman Chemical (Ann Arbor, MI). Omni-Solv™ acetonitrile and methanol purchased from EM Science (Gibbstown, NJ) and were used for all LC-MS/MS analyses. Other chemicals and supplies were obtained from Fisher Scientific (Pittsburgh, PA).

### Animals

Age matched male NIH Swiss mice weighing 25–30 g were obtained from The National Cancer Institute Animal Production Program at Frederick National Laboratory (Frederick, MD). A colony of sEH−/− mice with targeted deletion of sEH gene (*EPHX2*) which is backcrossed to C57BL/6 background was generated as described earlier [21]. The knockout and the con-specific wild type C57BL/6 mice were maintained at UC Davis. All experiments were performed using NIH Swiss mice except for those involving sEH−/− mice. Animals were housed in standard care facilities with a 12-hour light-dark cycle with free access to water and food.

### Treatments and behavioral tests

To investigate the effects of sEH inhibition on seizure behavior several standard acute tests involving the administration of pro-convulsant chemicals were employed as commonly described. For the subcutaneous picrotoxin (PIC) and pentylenetetrazol (PTZ) tests, 10 mg/kg PIC and 80 mg/kg of PTZ were administered by subcutaneous route, time to onset of first clonic seizure, time to tonic hind limb extension and lethality were monitored for a duration of 45 min for PIC and 30 min for PTZ. For the subcutaneous 4-aminopyridine test 13 mg/kg 4-AP was given subcutaneously, time to onset to tonic hind limb extension and lethality were monitored for 30 min. For the PTZ threshold test a 10 mg/mL solution of PTZ was infused via the tail vein of lightly restrained mice at a rate of 0.5 mL/min and the threshold to the onset of tonic extension was quantified. The threshold value (milligrams per kilogram) was determined according to the following formula: (infusion duration [seconds]×infusion rate [milliliters per minute]×convulsant drug concentration [milligrams per milliliter]×1000)/(60 s×weight of mouse [grams]). For the intraperitoneal (i.p.) PTZ seizure test, PTZ was administered by i.p route and parameters as outlined above were quantified. Two structurally different inhibitors of sEH were completely dissolved completely using PEG400 to give clear solutions as described earlier. Inhibitors or vehicle were administered subcutaneously 1 h prior to pro-convulsants at a range of doses. For the subcutaneous PTZ seizure test, diazepam injected in its own vehicle containing saline, 40% propylene glycol, 10% ethanol and the neurosteroid allopregnanalone (SAFC Pharma Inc, Madison, WI, USA) dissolved in PEG400 served as positive control. For the antagonism assay, finasteride was dissolved in PEG400 and administered 1 h prior to sEH inhibitor at a single dose of 10 mg/kg.

To investigate the direct roles of epoxy fatty acids the EETs, EpETEs and EpDPEs, diazepam (0.5 µg each), and sEH inhibitor TUPS (0.1 and 1 µg) were administered to mouse brain. Mice were anesthetized for 2 min in an induction chamber using isoflurane (3% with 2 LPM oxygen). Compounds were dissolved in vehicle (PEG400) and administered in a final volume of 1 µL into the cerebroventricular (i.c.v) space using a 22- gauge needle connected to a 10 µL hamilton syringe with a PE50 polyethylene tubing. The needle was removed 30 sec after injection was completed so as to prevent outflow and allow dispersion of the injected material. Mice fully recovered from anesthesia within 2 min indicated by responses to light touch and the presence of natural exploratory behavior. Thirty minutes following these treatments, i.p. PTZ (80 mg/kg) test was performed and seizure related parameters were quantified as described above.

### 6-Hz seizure test

Testing was carried out as previously described [22]. Briefly, 3-s corneal stimulation (200-µs duration, 32-mA monopolar rectangular pulses at 6 Hz) was delivered by a constant-current device (ECT Unit 5780; Ugo Basile, Comerio, Italy). Ocular anesthetic (0.5% tetracaine) was applied to the corneas 15 min before stimulation. Immediately before stimulation, the corneal electrodes were wetted with saline to provide electrical contact. The seizures were often preceded by a period of intense locomotor agitation (wild running and jumping). The animals then exhibited a “stunned” posture associated with rearing (bipedal standing), forelimb automatic movements and clonus, twitching of the vibrissae, and Straub-tail. The duration of the seizure activity ranged from 60 to 120 s in untreated animals. Animals resumed their normal exploratory behavior after the seizure. The experimental end point was protection against the seizure: an animal was considered to be protected if it resumed its normal exploratory behavior within 10 s of stimulation.

### Maximal electroshock (MES) seizure test

Animals were subjected to a 0.2-s, 60-Hz electrical stimulus through corneal electrodes. The electroshock unit was adjusted to deliver a constant current of 50 mA. Animals failing to show tonic hind limb extension were scored as protected [23].

### Tissue preparation and analyses

Inhibitor levels were quantified from blood and brain samples of treated animals using previously described methods [20]. Briefly, a 10 µL volume of cardiac blood sample was collected from each mouse post-mortem following the tonic seizure using a 27 Gauge needle. Blood was transferred into a tube containing 100 µL distilled water containing 1% EDTA, mixed immediately to prevent clots and stored at −80°C until further analysis. For quantification of brain inhibitor levels, brains were removed post-mortem following tonic seizures; a sample of tissue (∼50 mg) was taken from the prefrontal cortex area and stored at −80°C. The internal standard, compound APAU (1-(1-acetypiperidin-4-yl)-3-adamantanylurea, 250 ng/ml, 10 µL) along with 50 µL of distilled water was added to each thawed sample and tissues were broken down using a polypropylene microfuge pestle in 1.5 mL tubes. Extraction was then carried out three times with ethyl acetate (3×300 µL). The pooled supernatants of these extractions were dried and resuspended in 50 µl of methanol prior to analysis. The blood and brain levels of TUPS were determined as described previously using LC-MS/MS (Quattro Premier triple-quadrupole mass spectrometer, Waters, Milford, MA). The MS instrument was operated in positive electrospray ionization mode with selected reaction monitoring (SRM) following transitions: m/z 320.4>143 for compound APAU, and 382.3>169.4 for TUPS.

Eicosanoid analysis was performed on brain samples taken immediately following tonic seizures. Brains were removed; one hemisphere from each brain was rapidly frozen on dry ice, weighed and re-suspended in a solution containing 10 µL of anti-oxidants (0.2 mg/mL of BHT and EDTA), 10 µL of surrogate solution and 400 µL of ice-cold methanol with 0.1% of acetic acid and 0.1% of BHT. These samples were incubated at −80°C overnight for extraction. The next day, an internal standard solution (10 µL) containing deuterated standards was added and samples were homogenized using an ultrasonic-homogenizer at 30 Hz for 10 min and centrifuged at 10,000×*g* rpm for 10 min at 4°C. The supernatants were collected and pellets were re-extracted with 400 µL of ice-cold methanol with 0.1% of acetic acid and 0.1% of BHT and re-centrifuged. The supernatant of two extractions were combined and loaded onto preconditioned solid phase extraction cartridges (60 mg waters Oasis-HLB, Waters, Milford, MA), partially purified and reconstituted in 50 µl methanol as described previously. A 5 µL aliquot was directly analyzed by LC-ESI-MS/MS as described previously. The limit of quantification (LOQ) of the method was defined as the quantity of sample required to produce a signal-to-noise ratio (S/N) of at least 10. Typically calibration standards had LOQs between 0.07 and 32 pg for the deuteriated analytes as reported earlier.

### Statistical Analyses

Data were analyzed by non-parametric and parametric One way-ANOVA followed by appropriate post hoc tests for between group comparisons selected by the software suite SigmaPlot analysis package (Systat Software, Inc., Chicago, IL). Results are presented as mean ± standard error of mean.

## Results

### sEH−/− mice have delayed seizures in response to GABA antagonists

**Figure 1:**
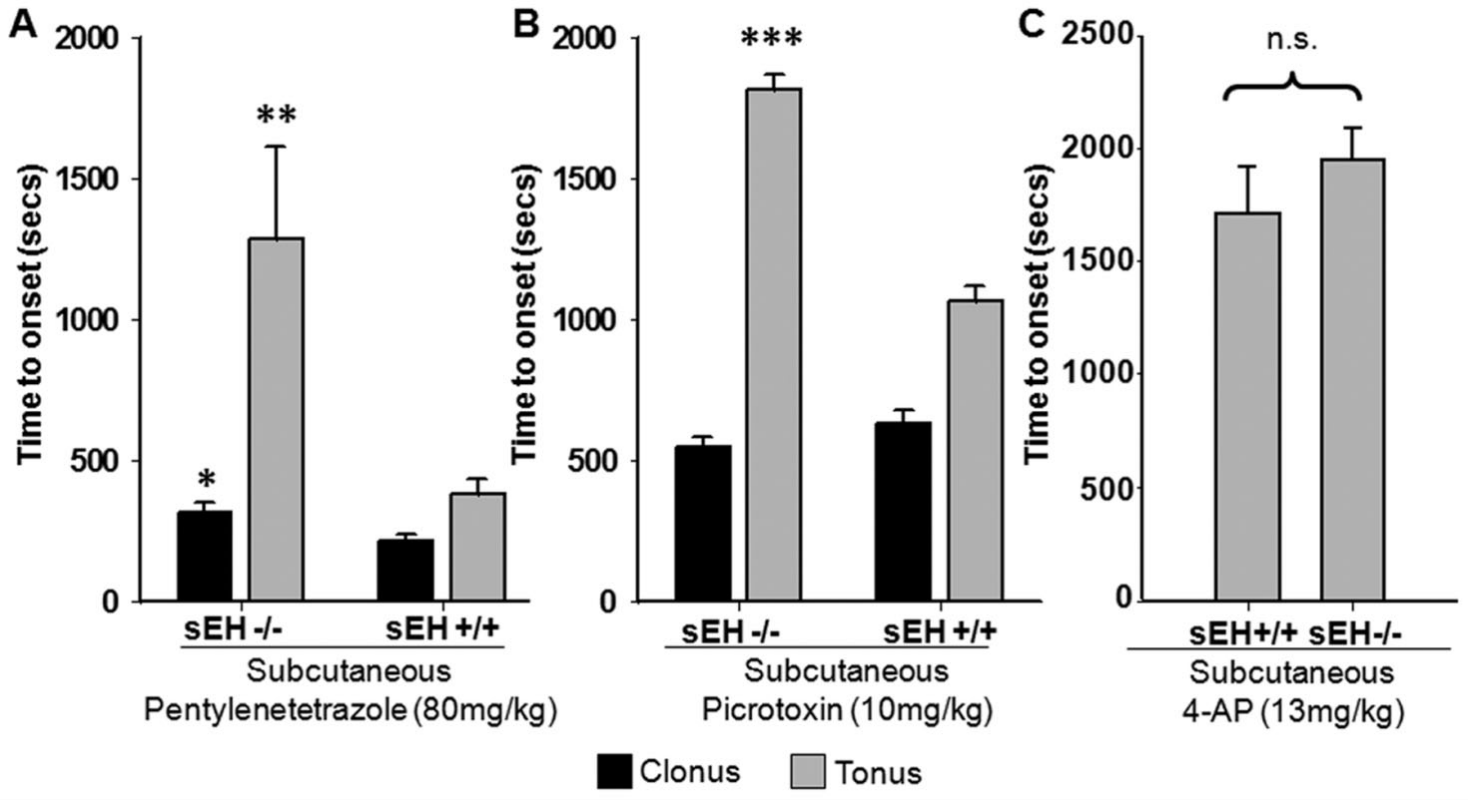
Genetic deletion of sEH delays seizure onset induced by GABA antagonists. Deletion of the sEH gene in mice stabilizes EETs and delays onset of seizures induced by pentylenetetrazole (PTZ) and picrotoxin (PIC). (*A*) Subcutaneous PTZ led to a set of stereotypical convulsive behaviors including myoclonic (black bars) and tonic (gray bars) seizures in mice. In sEH−/− mice both clonic and tonic phase of seizures were delayed compared to conspecific C57/BL6 mice (n = 6 mice/group, Student's *t*-test, sEH−/− vs. wt, *p = 0.049 and **p = 0.001). Note that animals that did not display tonic hind limb extension reflex within 30 min were excluded from these graphs (see Table 1 for survival data). (*B*) Onset of tonic seizures induced by subcutaneous PIC were delayed in sEH−/− mice (n = 5 mice/group, Student's *t*-test, sEH−/− vs. wt, ***p = 0.003), while onset of PIC induced clonic seizures did not differ (p = 0.75). (*C*) Seizures induced by 4-AP led to tonic seizure without a clear clonic phase and onset in sEH−/− and wt mice were not significantly different (n = 7/group, p = 0.39). Data are expressed as mean ± s.e.m for all figures.

Several prior observations led to the hypothesis that at least part of the analgesic effects of EpFAs and inhibitors of sEH may be through a GABA mediated mechanism [3], [12]. To test the hypothesis that EpFAs and sEH are influence GABA-ergic signaling, subcutaneous PIC and PTZ tests were performed in sEH−/− and sEH+/+ conspecific mice. In both tests delayed onset of tonic hind limb extension and reduced lethality were observed with sEH−/− mice compared to wild type conspecific mice (**Fig. 1A and 1B**). Notably, there was a marginal, though significant, delay in time to onset of clonic seizures in the PTZ treated sEH−/− mice not observed with PIC. Even though onset to clonic seizures was not largely affected, in sEH−/− mice delayed lethality was accompanied with lack of tonic hind limb extension reflex, supportive of the role of EpFAs in modulating CNS function. The difference in responses to PIC and PTZ may be based on the fact that their binding sites on the GABA_A_ complex are overlapping but not identical [24]. In contrast to the GABA antagonist initiated seizures, the non-selective voltage gated potassium channel blocker 4-AP induced seizures had identical potency in wild type and sEH−/− mice, highly suggestive of a GABA mediated mechanism of action for sEH deletion (**Fig. 1C**).

### Potent sEH inhibitors delay onset of GABA antagonist elicited tonic seizures

**Figure 2:**
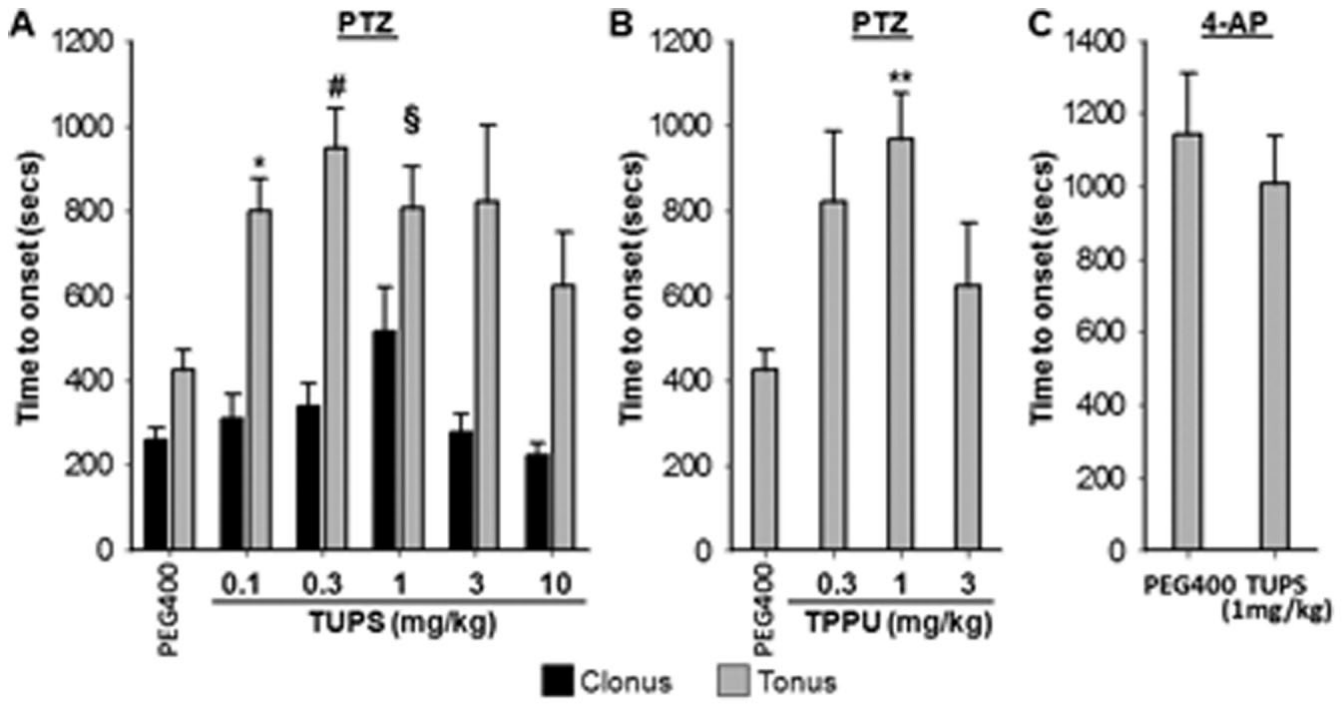
Potent inhibitors of sEH dose dependently delay onset of seizures induced by GABA antagonist. In parallel to observations with sEH−/− mice, small molecule inhibitors of sEH delay onset of tonic phase of seizure in wild type Swiss mice. In all three experiments sEHI were administered one hour prior to convulsants to allow absorption and distribution of the inhibitor. (*A*) TUPS was administered at a volume of 1 µL/g body weight by intraperitoneal route following completely dissolving it in PEG400. Subcutaneous PTZ induced tonic seizure is delayed by sEH inhibitor TUPS (n = 8–22/group, Kruskal-Wallis One Way ANOVA on Ranks followed by Dunn's multiple comparison, vehicle vs TUPS, *p = 0.028, **#** p = 0.008, **§** p = 0.03), though onset of clonic seizure was not affected by inhibition of sEH. (*B*) In parallel to results obtained by TUPS, a structurally different sEHI, TPPU delayed the onset of tonic phase of PTZ induced seizure compared to vehicle (n = 8–14/group, Kruskal-Wallis One Way ANOVA on Ranks followed by Dunn's multiple comparison, vehicle vs TPPU, **p≤0.05). Clonic phase of seizures was not monitored in this experiment. See Table S1 for structure and potency information of TUPS and TPPU. (*C*) Seizures induced by 4-AP had a shorter onset in Swiss mice however consistent with data in Figure 1, an efficacious dose of TUPS did not significantly alter onset of seizure (n = 8/group, Student's *t*-test, p = 0.49).

These observations prompted us to investigate if a comparable phenotype could be recapitulated by using potent small molecule inhibitors of sEH. Dose dependent effects of two structurally different but highly potent inhibitors TUPS and TPPU were studied and more detailed characterization was carried out using TUPS. In the subcutaneous PTZ assay, both inhibitors significantly delayed the onset of tonic hind limb extension and reduced lethality when administered 1 h prior to PTZ by intraperitoneal (i.p.) route (**Fig. 2** **and** **Table 1**). Note that animals that were protected from tonic hind limb extension during the 30 min observation time are excluded from these graphs and presented in Table 1. Time to onset of clonic seizure was not affected by inhibition of sEH (**Fig. 2A**). In parallel to the observations with sEH−/− mice tonic seizures induced by 4-AP were not different between vehicle and TUPS treated mice, though the Swiss mice were more sensitive to 4-AP (**Fig. 2C**). In low dose groups sEHI significantly delayed the onset of seizures, though at higher doses efficacy was lower. Nevertheless, mice treated with TUPS were protected from tonic seizures in a linear and dose dependent manner (**Table 1**). Compared to an effective dose of diazepam which prevented clonic seizures in 50% and tonic seizures in 100% of the mice, TUPS protected 44% of the mice from tonic hind limb extension (**Table 1**). Unlike the sEH−/− mice, tonic hind limb extension was a consistent phenotype in animals pretreated with both sEHI.

**Table 1:** The sEHI treatment reduced the proportion of mice experiencing tonic seizures but did not provide significant protection against clonic seizures.

compound (mg/kg)+pentylenetetrazole	mean§ time to clonic seizure in seconds (SEM)	protected from clonic/total	mean§ time to tonic seizure in seconds (SEM)	protected from tonic/total
Vehicle (PEG400)	**257 (31)**	**0/12**	**425 (45)**	**0/12**
TUPS (0.1)	**308 (59)**	**0/14**	799 (77)*	**0/14**
TUPS (0.3)	**338 (54)**	**0/22**	947 (94)*	7/22*
TUPS (1)	**516 (102)**	**0/18**	809 (95)*	8/18*
Diazepam (0.3)	**192 (57)**	**0/8**	633 (140)*	**0/8**
Diazepam (1)	**404 (21)**	4/8*	**>1800**	8/8*

Diazepam at a high dose was effective in protecting from both clonic and tonic seizures.

^§^Mean values with animals protected from clonic or tonic seizures excluded.

**Figure 3:**
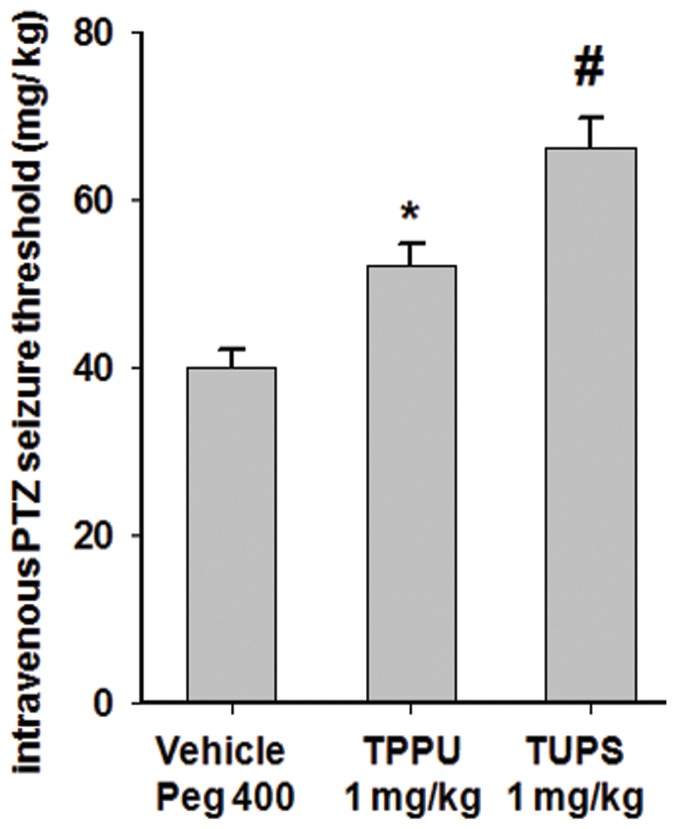
Potent inhibitors of sEH elevate threshold of tonic seizure in the timed i.v. infusion test. Consistent with the subcutaneous PTZ test both TUPS and TPPU, administered 1(vehicle n = 8, TPPU n = 6, TUPS n = 3, One Way ANOVA followed by Student Newman Keuls post hoc analysis *p = 0.026, **#** p = 0.005).

Next, PTZ threshold test was performed to ask if stabilization of EpFAs by pretreatment with sEHI would change acute seizure threshold. The threshold test is highly consistent and eliminates the potential confounding effects of the pharmacokinetics of PTZ, thus is used for comparison of the anticonvulsant potency of therapeutic candidates. Pre-treatment with two structurally different sEHI resulted in significant increase in threshold for tonic hind limb extension for both compounds supporting the earlier observations (**Fig. 3**). In the maximal electric shock and 6 Hz seizure tests, TUPS (i.p. administration, 1 mg/kg, n = 8) and TPPU (i.p. administration, 1 mg/kg, n = 8) displayed no observable effect.

### Effects of sEH inhibitors are partially blocked by finasteride and enhanced by allopregnanolone

Earlier data on anti-nociceptive effects of sEH inhibitors suggested that at least part of the analgesic activity could be mediated by a neurosteroid mediated mechanism [3]. To test if the effects observed here are mediated by a potential effect on neurosteroids we used a two pronged approach; brain permeable steroid synthesis inhibitor finasteride was tested for antagonism and allopregnanolone, a major neurosteroid, was tested for synergy. The efficacy of TUPS in delaying tonic seizure onset was partially blocked by pretreatment with an effective dose of finasteride (1 h prior to sEHI). However, finasteride had no effect on the ability of sEHI to protect from tonic seizures (**Table 2**). In addition, finasteride significantly reduced the marginal delay in the onset of clonic seizures elicited by sEHI. Moreover co-administration of TUPS with an ineffective dose of allopregnanalone resulted in an enhanced efficacy in both, delaying the onset of tonic seizures and protection from tonic hind limb extension. These data argue that a sEHI and EETs may have an interaction with neurosteroid(s) or GABA-related signalling.

**Table 2:** The effects of allopregnenolone was significantly enhanced by sEHI and partially antagonized by finasteride (One Way ANOVA followed by Student Newman Keuls post hoc analysis TUPS+Allo vs TUPS, *p = 0.016, TUPS+Allo vs Allo, **p = 0.007, TUPS vs TUPS+FIN, #p = 0.02).

compound (mg/kg)+pentylenetetrazole	mean time to clonic seizure in seconds (SEM)	mean time to tonic seizure in seconds (SEM)§	protected from tonic/total
Vehicle (PEG400)	**257 (31)**	**425 (45)**	**0/12**
TUPS (1)	**516 (102)**	809 (95)*	**8/18**
Finasteride (10)	**204 (38)**	**619 (80)**	**0/8**
TUPS+Finasteride (1+10)	**185 (19)#**	**630 (66)**	**3/8**
Allopregnenolone (1)	**161 (24)**	587 (145)**	**0/4**
Allopregnenolone (3)	**397 (16)**	**850 (40)**	**1/4**
TUPS+Allopregnenolone (1+1)	**537 (113)**	**1663 (478)**	**3/4**

Finasteride was administered 2 h, TUPS was administered 1 h, allopregnenolone, 10 min prior to PTZ.

^§^Observation period was extended to 45 min to increase the dynamic range of the assay.

### Inhibition of sEH elevates brain levels of EpFAs but does not alter the seizure elicited increases in PGE_2_ and PGD_2_

**Figure 4:**
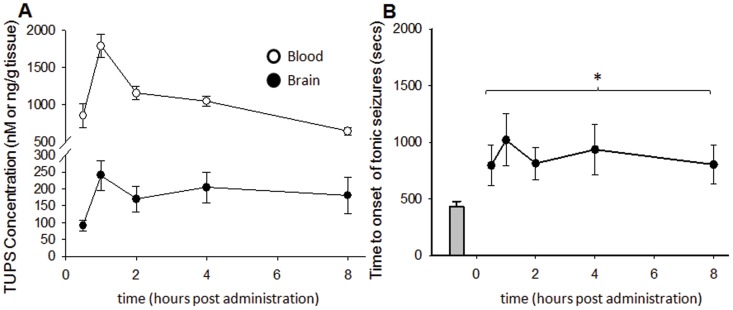
Brain levels of sEHI correlate with observed activity. The sEHI TUPS was administered at a single i.p. dose of 1/kg and seizures were induced at varying times using s.c. PTZ procedure (n = 8/time point). Following tonic hind limb extension and lethality, brains were rapidly excised and TUPS was quantified by LC-MS/MS. Protected animals (3 of 8 in each group) were sacrificed 30 min following PTZ treatment and are included on the left panel. (A) TUPS was well absorbed and blood and brain inhibitor levels reached well over in vitro IC_50_ values within an hour and remained relatively stable over 8 h. (B) The efficacy of TUPS was significantly correlated with brain inhibitor levels (Spearman's Rho, 0.90, p = 0.037) All time points are significantly different from the vehicle treated animals (One Way ANOVA, p = 0.034).

The potent sEHI TUPS is a brain permeable inhibitor in the rat [12]. Here we asked if TUPS was present in the mouse brain and if blood or brain levels of the inhibitor correlated with the observed delays in the onset of seizures. TUPS was rapidly absorbed and blood inhibitor levels reached µM concentration within an hour (**Fig. 4**). Brain concentration of TUPS reached over 200 nM within one hour and remained little changed within the 8 h period. Even though the blood to brain ratio shifted from 9.3 to 3.5 over the time course of the assay, the quantities measured in the blood and brain were both well over the *in vitro* IC_50_ value of TUPS (5 nM, using recombinant mouse sEH and the fluorescent CMNPC assay) throughout the 8 h observation period (**Fig. 4**).

**Figure 5:**
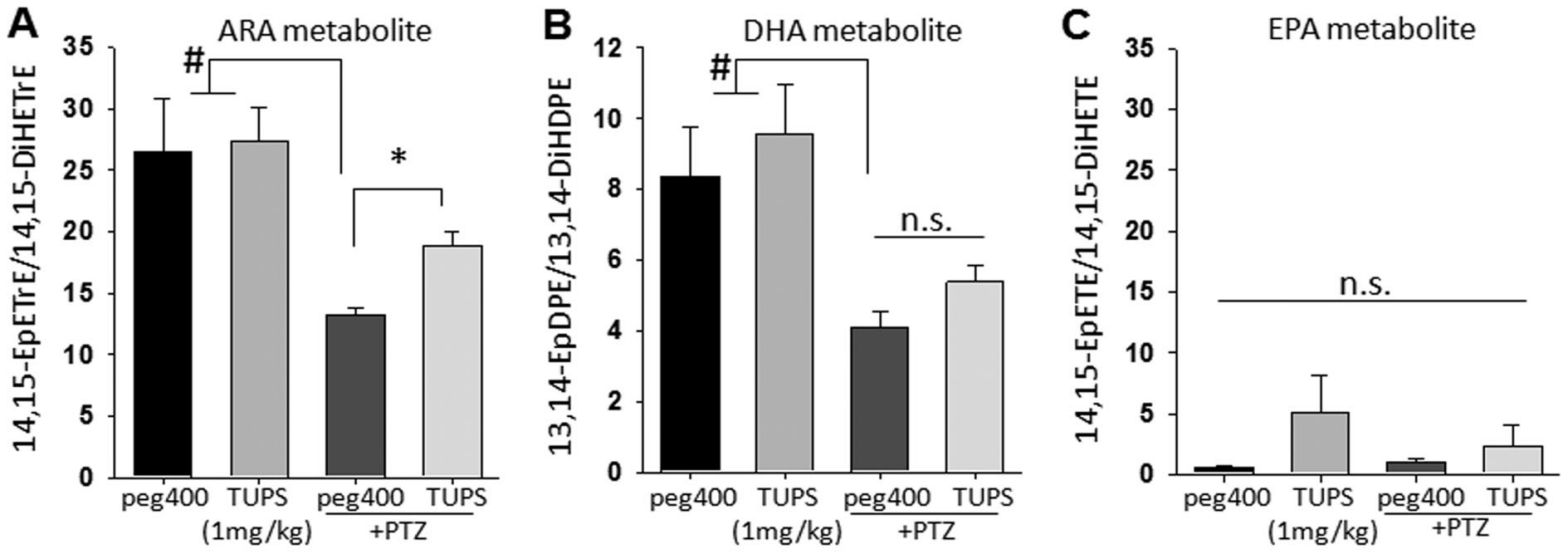
Brain oxylipins display decrease in response to seizure and are elevated by sEHI. (*A–C*) Bar graph of ratios of brain epoxy to dihydroxy-FAs in mice receiving vehicle (n = 6, black bars), sEHI (n = 6, gray bars), vehicle+PTZ (n = 8, dark gray bars) and sEHI+PTZ (n = 8, light gray bars). Inhibitors were administered 1 h prior to sampling, PTZ treated animals were sampled up immediately following tonic phase of the seizure. PTZ treatment resulted in a significant decrease in the levels of bioactive regioisomers 14,15-EET and 13,14- EpDPE (Kruskal-Wallis One Way ANOVA on Ranks followed by Dunn's multiple comparison, control vs PTZ, **#** p≤0.05). Although in the absence of seizures sEHI did not lead to changes in the brain levels of 14,15- EET and 13,14- EpDPE, the decrease mediated by seizures was recovered by inhibition of sEH for the ARA metabolite 14,15-EET (*p = 0.001) but not for the DHA metabolite 13,14-EpDPE (p = 0.061). There were no significant changes in the levels of EPA derived epoxy fatty acids among groups.

Next we asked if inhibition of sEH in the brain, the site of action for anticonvulsant drugs, modulated the levels of bioactive lipids. Brains were excised immediately following tonic seizures or 30 min following i.p. saline under deep anesthesia for control groups, frozen on dry ice extracted on the same day and stored until analysis. The ratio of epoxy- to dihydroxy- eicosanoids (epoxide to diol ratio) is used as a marker of sEH inhibition *in vivo* [12]. The high levels of inhibitor in brain tissue coincided with significant but selective increases in the levels of the EETs (**Fig. S1**). Although the levels of the bioactive regioisomer, 14,15-EET, did not display a significant change in response to the inhibitor, it was significantly diminished by seizure activity and recovered in PTZ exposed and sEHI treated seized mice (**Fig. 5A**). With the exception of the epoxide on the omega-3 olefins, the epoxides of EPA and DHA are better substrates for the sEH than the corresponding epoxide regioisomers of ARA [4]. These are elevated by sEHI in the plasma and are active in assays of pain. Here, the lack of response in epoxy-EPA and epoxy-DHA regioisomers to sEH inhibition was remarkable and could stem from other regulatory mechanisms such as synthesis, membrane incorporation or re-incorporation (**Fig 5B, C** **and Fig. S1**). The different responses of various EpFAs to sEHI and seizures suggest distinct regulatory mechanisms for individual lipid metabolites.

Consistent with previous observations there was a general increase in the brain levels of major prostanoids in wild type and sEH−/− mice following tonic seizures induced by PTZ and 4-AP (**Fig. S2**). Although the role of each prostanoid in seizures is not clear, in general there is a consensus about pro-convulsant or seizure enhancing role of PGE_2_, while PGD_2_ is thought to have the opposite effect. Regardless, inhibition of sEH by genetic knockout or by sEHI resulted in a highly consistent profile of increased brain levels of PGE_2_ and PGD_2_ which was not reduced in sEH−/− or sEHI treated animals (**Fig. S2**). These results demonstrate that the effect of sEH inhibition is independent of prostanoid levels. These findings are contrary to the extensive effects of sEHI on prostanoid levels in models of peripheral inflammation [9]. Thus here a distinct mechanism for anti-convulsant effect of inhibition of sEH is demonstrated.

### EETs and sEHI but not epoxy-DHA or epoxy-EPA metabolites administered into the brain delay onset of PTZ induced seizures

**Figure 6:**
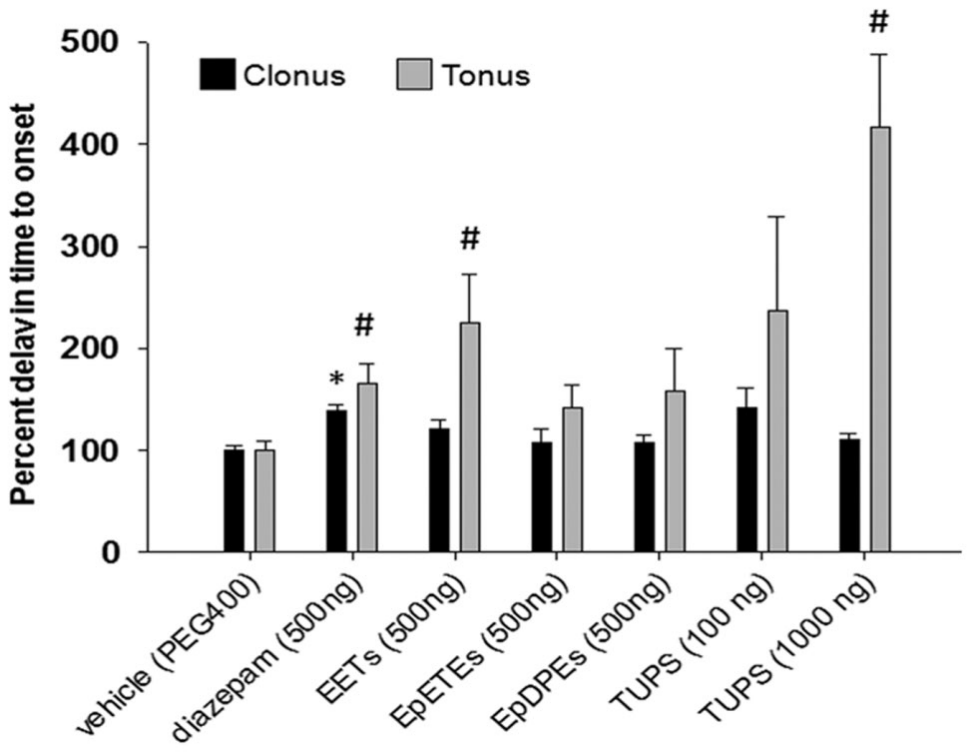
Direct administration of EETs and sEHI into the brain delay onset of PTZ induced seizures. Bar graph of percent change in onset of clonic (black bars) and tonic (gray bars) seizures following intracerebroventricular administration of EpFAs, sEHI and diazepam (n = 6–14mice/group). Methyl ester EpFAs, sEHI and diazepam were dissolved in PEG400 and 1 µL was given by i.c.v. route to lightly anesthesized mice in the amounts indicated on *x-axis*. Thirty minutes after treatments PTZ assay was performed. Among the treatment groups, only diazepam was effective in delaying the onset of clonic seizures (Kruskal-Wallis One Way ANOVA on Ranks followed by Dunn's multiple comparison, *p≤0.05). Diazepam, EET-methyl esters and high dose of sEHI and were effective in delaying the onset of tonic seizures (Kruskal-Wallis One Way ANOVA on Ranks followed by Dunn's multiple comparison, **p≤0.05).

If inhibition of sEH is anticonvulsant by elevating the brain levels of EpFAs then direct administration of EpFAs and sEHI into the brain should also delay the onset of seizures. Therefore we tested anticonvulsant effects of direct administration of EpFAs and sEHI into the mouse brain. Mice were lightly anesthetized, compounds delivered by i.c.v. route in 1 uL volume, and PTZ (80 mg/kg) administered i.p. 30 min after i.c.v. injection. A low dose of diazepam was used as a positive control. Consistent with the changes in the brain levels of EpFAs, only the regioisomeric mixture of EET methyl esters (epoxy-ARA metabolites) but not EpDPE or EpETE methyl esters delayed the onset of clonic and tonic seizures (**Fig. 6**). The efficacy of EETs on tonic seizures was equivalent to that of diazepam. Both the high and low dose of TUPS was significantly effective in delaying seizure onset. Compared to diazepam, the high dose of TUPS (1 µg) was significantly more active. In pilot experiments similar results were obtained with EETs and TUPS 10 min following i.c.v. administration, although the 30 min post i.c.v. paradigm was preferred in order to minimize the influence of anesthetics on the assay (**Fig. S3**). These data overall demonstrate that the majority of the biological activity of sEH inhibitors is mediated by elevation of EETs in the brain.

## Discussion

The levels of the bioactive EpFAs are controlled by several distinct mechanisms [19], [25]. Independently, intracellular concentration of the free EpFAs are tightly regulated by the enzyme soluble epoxide hydrolase (sEH) which converts the EpFAs to their corresponding diols, molecules that are inactive or in some cases seem to have pro-inflammatory roles or effects on development [10], [26]. Overall, the CNS tissues possess the capacity to synthesize EpFAs *de novo* and rapidly degrade these molecules via sEH activity. Region specific existence of various EpFAs and sEH in the CNS has also been reported [27], [28]. Augmenting levels of EpFAs by affecting these two independent regulatory mechanisms, by enhancing the release from membrane stores through cAMP mediated lipolysis and by inhibiting sEH activity, suppress pain related behavior in rodents and horses under acute, inflammatory and neuropathic paradigms [3], [12], [13], [29]. Thus, understanding the mechanism of action of these novel mediators from both the fundamental and applied perspectives is of interest since this system can be therapeutically exploited for the treatment of a diverse number of neurological disorders including neuropathic pain.

A number of structurally different but potent small molecule inhibitors of sEH have been demonstrated to stabilize the EpFAs *in vivo*, strongly indicating that the mechanism of action of sEHI is through stabilization and prolonging the activity of the EpFAs [9], [13]. Moreover, promoting the release of EpFAs through a cAMP mediated pathway and concurrently inhibiting their metabolism by sEHI results in synergistic and extended duration of activity in assays of pain [12]. Here, increases in the levels of EpFAs either through deletion of the sEH gene, inhibition by potent small molecule inhibitors or by exogenous administration into the brain produced matching results. These data support the idea that the mechanism of action of sEH inhibitors is through stabilization of EpFAs. Although the urea, amide or carbamate inhibitors of sEH may have other effects than inhibiting sEH, and sEH−/− mice may exhibit compensatory mechanisms, here we demonstrate that observed delays in onset of seizures are mediated by EpFAs, and specifically EETs.

The finding that convulsions produced by GABA_A_ antagonists PIC and PTZ are impeded by inhibition or deletion of sEH is of significance. Equally important, these effects seem to be selective towards GABA_A_ receptors. Given that 4-AP induced seizures were consistently unaffected by inhibition of sEH, the observed effects should not be driven by the potential ability of EETs to modulate BK_Ca_ mediated hyperpolarization in neurons [30]. Earlier findings describing a GABA_A_-mediated mechanism of action in the antihyperalgesic effects of EpFAs and sEHI are in agreement with this study [3], [12]. Moreover, results here demonstrate a direct effect of EpFAs and specifically EETs on suppression of excitatory signaling in the brain, which could at least partially be the basis of the antihyperalgesic effects of EpFAs. Notably, comparable increase in the PTZ threshold test support the anticonvulsant effects of positively modulating EpFAs.

Although it is unclear if EpFAs enhance neurosteroid synthesis and thus enhance GABA-ergic inhibition, more evidence towards the presence of an interaction between EpFAs and the GABA system is presented here. More to the point, finasteride, a prototypical inhibitor of 5α-reductase partially blocked the effects of sEHI, while allopregnanolone enhanced these effects. Importantly, these results are similar to those obtained in pain assays suggesting a common mechanism of sEHI mediated suppression of neuronal excitability.

Given the effects mediated by sEH inhibition in assays of seizures one would expect to see corresponding changes in the levels of EpFAs in the brain, the site of convulsions. Our analysis of mouse brain demonstrates expected changes in the brain levels of EpFAs in response to sEH inhibition. Nevertheless, it was surprising to observe highly selective, regioisomer specific changes in the levels of EpFAs. Although metabolites of ARA, including prostanoids were generally increased the bioactive regioisomer 14,15-EET was reduced along with all DHA derived EpFAs, yet EPA derived EpFAs were elevated after seizures. The basis of these changes were not addressed in this study, though these observations justify further work to enhance our understanding of mechanisms regulating free fatty acids and their metabolites in the brain. Ictal activity leads to the release of ARA from membrane stores [31], [32]. This free acid form is a potential substrate for cyclooxygenase, lipoxygenase and cytochrome P450 enzymes which convert ARA to prostanoids, leukotrienes, EpFAs and other metabolites such as the 19-HETE and 20-HETE which are another group of cytochrome P450 generated omega hydroxyl metabolites [5], [33]. Consistent with this knowledge, we observed increased levels of prostanoids in the brain following seizures induced either with PTZ or 4-AP (**Fig. S2**). These increases in prostanoids validate our LC-MS/MS based approach. However, surprisingly the changes were asymmetric among the products of the three branches of the ARA cascade, with increases only in prostanoids and variable levels in cytochrome P450 products. This indicates a degree of dynamic regulation in the synthesis of bioactive lipids in the brain. An advantage of our approach is that all metabolites are analyzed together, thus a more comprehensive view of the biological processes is obtained. A limitation of this study however is that one hemisphere of the brain was analyzed, thus we are unable to determine the loci of the changes. Levels of EETs vary by brain region, therefore our results likely underestimate local changes in response to sEH inhibition [34].

In contrast to earlier findings in acute inflammatory models in which inhibition of sEH leads to suppression of transcriptional upregulation of COX-2 and consistently reduces the levels of major prostanoids, here genetic or chemical inhibition of sEH had no effect on the levels of brain PGE_2_ and PGD_2_. This observation adds to the argument that fundamentally different mechanisms underlie anticonvulsant and anti-inflammatory activities of sEH inhibition.

Lastly, these results encouraged us to test EpFAs directly by i.c.v. administration. The regioisomeric mixture of cell permeable EET-methyl esters were exclusively active in delaying onset of tonic seizures while the epoxy-DHA and -EPA metabolites were largely devoid of effects in the brain. The parallel results obtained with EETs and sEHI following i.c.v. administration are consistent with that of systemic sEHI and experiments using sEH−/− mice. The EETs were as potent as the low dose of diazepam in this case, though their intrinsic activity is likely to be lower than that of an effective dose of diazepam. Data from this study suggests that more effort into understanding the effects of EETs in the brain is warranted and will likely yield valuable information on how EETs and other EpFAs modulate physiology of the brain, particularly under excitatory conditions. In particular presence of sufficient amounts of EpFAs may prevent synchronized discharges as suggested by the observation that in sEH−/− and i.c.v. EET treated mice, tonic extension reflex was not consistently observed. An interesting future direction, and a limitation of this study, is the quantification of EpFAs following clonic seizures to determine if the reduction in the levels of EpFAs is because of clonic or tonic seizures.

EpFAs and sEH inhibitors display numerous positive effects in multiple disease models. Here we demonstrate yet another mechanism of action for EpFAs that has not been shown previously. Although molecular target(s) for EpFAs are not yet identified, the structural diversity of EpFAs that are stabilized by preventing their degradation may, based on concentration and context, lead to modulation of different receptors. In this case, a clear effect on GABA mediated excitotoxicity is linked with only one group of EpFAs, the EETs. This type of selectivity also argues that sEH inhibition leads to context based pleiotropic effects because one modulates a diverse set of EpFAs with sEHI.

Even though rodent models of convulsions are considered to be clinically relevant, an estimated one third of patients with epilepsy are thought to suffer from intractable or pharmaco-resistant epilepsy [1], [35]. This rate of prevalence underscores the complexity of the conditions that fall under the umbrella term of epilepsy. The sEHI may have clinical utility in this regard. The unique profile of activity of sEHI and EETs on convulsive models paves the way to the investigation of the EET signaling system in a wide spectrum of epileptic disorders. The current potent sEH inhibitors now provide the pharmacological tools required for the exploration of the role of the EpFAs in CNS function. As the knowledge of EpFA signaling in the brain advances and brain selective sEHI become available, it may be possible to target a clinical subpopulation in which sEH inhibition provides therapeutic benefit.

## Supporting Information

Figure S1 **Brain levels of major EpFAs and their corresponding dihydroxy- metabolites change after PTZ induced seizure.** Following tonic hind limb extension the brain levels of sum of EpFAs and their corresponding dihydroxy- metabolites from ARA (8,9-EET, 11,12-EET and 14,15 EET), DHA (10,11-EpDPE, 13,14-EpDPE, 16,17- EpDPE and 19,20-EpDPE) and EPA (8,9-EpETE, 11,12-EpETE, 14,15-EpETE and 17,18-EpETE) showed distinct patterns indicative of sEH dependent and independent mechanisms that selectively regulates their levels. (A) The sum of EET regioisomers remained unchanged in response to seizure but were elevated by inhibition of sEH with a concomitant decrease in the sum of corresponding dihydroxy- metabolites. (B) The sum of EpDPE regioisomers were decreased following seizures but were unchanged in response to inhibition of sEH. (C) The sum of EpETE regioisomers and their corresponding dihydroxy metabolites increased in response to seizure. While inhibition of sEH did not further elevate the EpETEs TUPS reduced the levels of the corresponding dihydroxy metabolites.
(TIF)

Figure S2 **Brain levels of major prostanoids increase following tonic hind limb extension induced by PTZ and 4-AP.** (A) In wild type and sEH−/− mice brain levels of major prostanoids display a highly similar profile in which PGE_2_, PGD_2_ and PGF_2α_ and PGJ_2_ are elevated to the same degree. This data suggests brain sEH had no role in modulating the levels of prostanoids in the brain after seizures. (B) Parallel results are obtained with small molecule inhibitor of sEH. (C) Seizure induced by 4-AP also led to an increase in prostanoids although these increases were smaller in magnitude than PTZ induced changes.
(TIF)

Figure S3 **Direct administration of EETs and sEHI into the brain delay onset of PTZ induced seizures.** Bar graph of percent change in onset of clonic (black bars) and tonic (gray bars) seizures following intracerebroventricular EETs and TUPS (n = 6–14mice/group) 10 min post administration (see Fig. 6 for 30 min post i.c.v. administration). Even though the profile of EpFAs were studied at 30 min post ic.v. dosing, a small pilot experiment was performed by testing PTZ 10 min after ic.v. administration. In this assay the regioisomeric mixture of EET methyl esters as well as the sEHI were efficacious in delaying the onset of seizures (Kruskal-Wallis One Way ANOVA on Ranks followed by Dunn's multiple comparison, clonus *p≤0.05, tonus #p≤0.05).
(TIF)

Table S1 **Structure and properties of sEHI used in the study.**
(TIF)
