# Supplementary figures and images for: Epoxy Fatty Acids and Inhibition of the Soluble Epoxide Hydrolase Selectively Modulate GABA Mediated Neurotransmission to Delay Onset of Seizures
Source: PLoS One. 2013 Dec 11;8(12):e80922. doi: 10.1371/journal.pone.0080922 (PMC3862847; doi:10.1371/journal.pone.0080922)

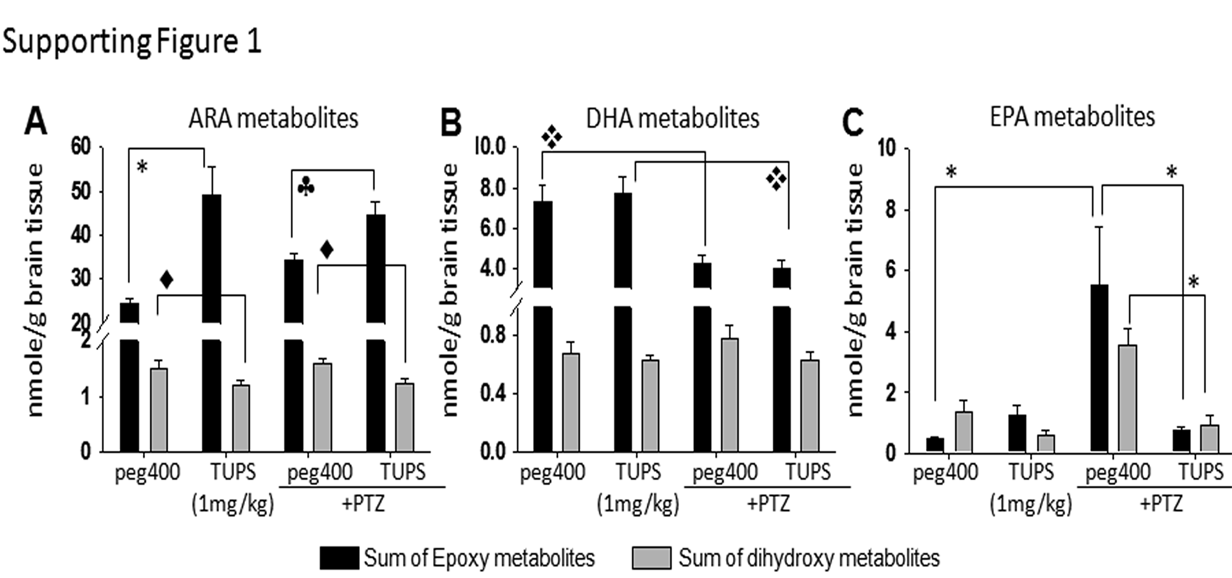

Supplement: Figure S1 — Brain levels of major EpFAs and their corresponding dihydroxy- metabolites change after PTZ induced seizure. Following tonic hind limb extension the brain levels of sum of EpFAs and their corresponding dihydroxy- metabolites from ARA (8,9-EET, 11,12-EET and 14,15 EET), DHA (10,11-EpDPE, 13,14-EpDPE, 16,17- EpDPE and 19,20-EpDPE) and EPA (8,9-EpETE, 11,12-EpETE, 14,15-EpETE and 17,18-EpETE) showed distinct patterns indicative of sEH dependent and independent mechanisms that selectively regulates their levels. (A) The sum of EET regioisomers remained unchanged in response to seizure but were elevated by inhibition of sEH with a concomitant decrease in the sum of corresponding dihydroxy- metabolites. (B) The sum of EpDPE regioisomers were decreased following seizures but were unchanged in response to inhibition of sEH. (C) The sum of EpETE regioisomers and their corresponding dihydroxy metabolites increased in response to seizure. While inhibition of sEH did not further elevate the EpETEs TUPS reduced the levels of the corresponding dihydroxy metabolites. (TIF) [file pone.0080922.s001.tif]

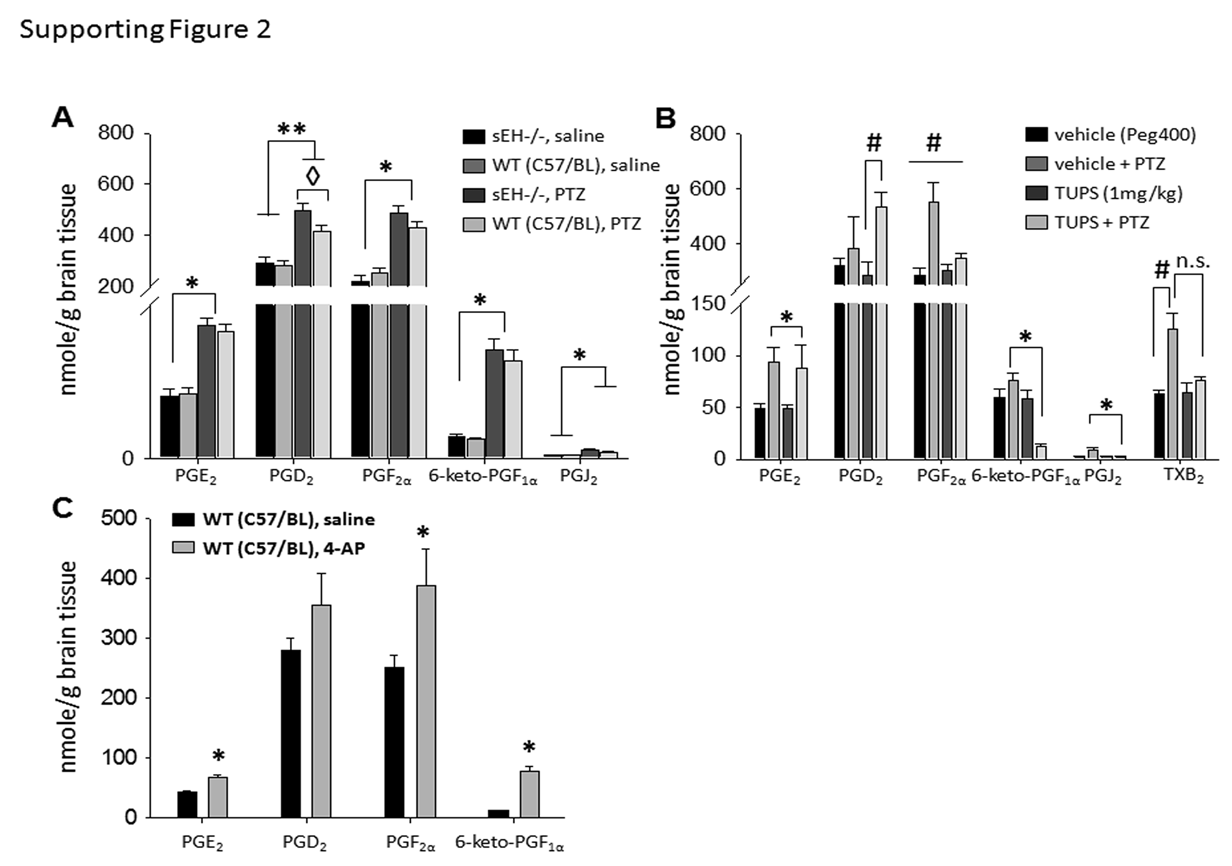

Supplement: Figure S2 — Brain levels of major prostanoids increase following tonic hind limb extension induced by PTZ and 4-AP. (A) In wild type and sEH−/− mice brain levels of major prostanoids display a highly similar profile in which PGE2, PGD2 and PGF2α and PGJ2 are elevated to the same degree. This data suggests brain sEH had no role in modulating the levels of prostanoids in the brain after seizures. (B) Parallel results are obtained with small molecule inhibitor of sEH. (C) Seizure induced by 4-AP also led to an increase in prostanoids although these increases were smaller in magnitude than PTZ induced changes. (TIF) [file pone.0080922.s002.tif]

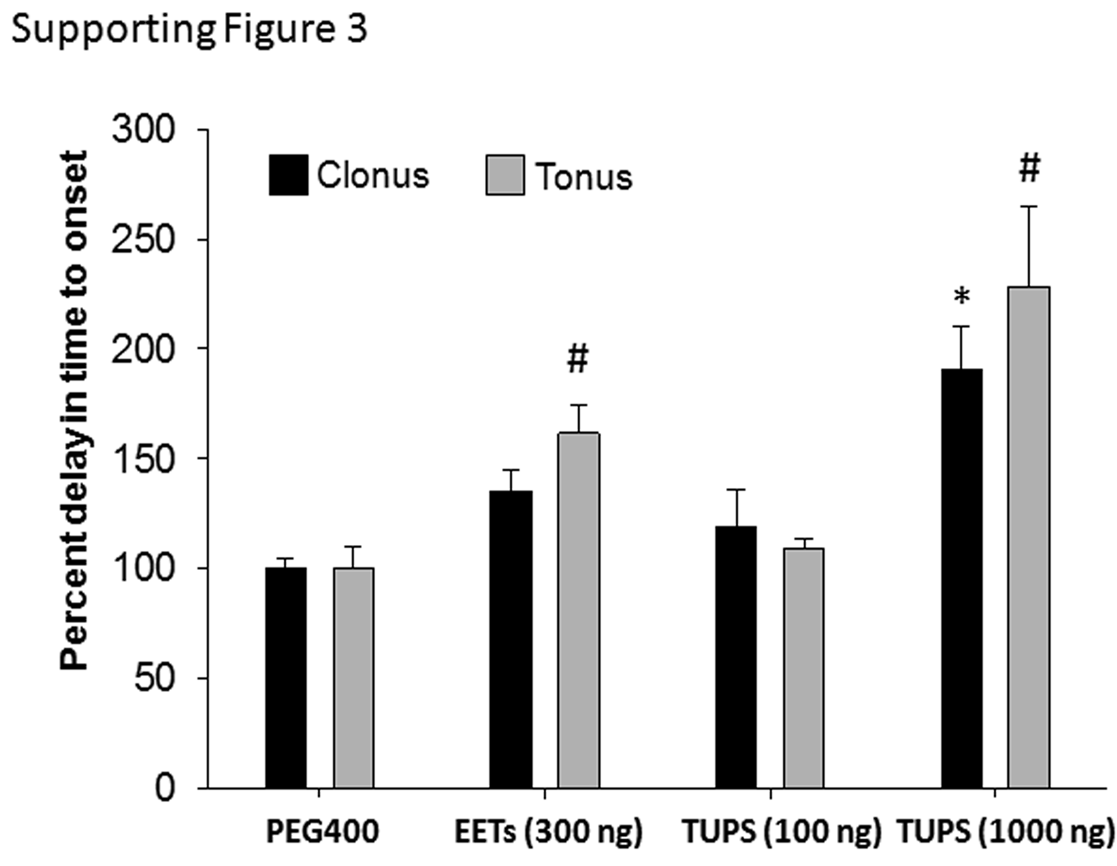

Supplement: Figure S3 — Direct administration of EETs and sEHI into the brain delay onset of PTZ induced seizures. Bar graph of percent change in onset of clonic (black bars) and tonic (gray bars) seizures following intracerebroventricular EETs and TUPS (n = 6–14mice/group) 10 min post administration (see Fig. 6 for 30 min post i.c.v. administration). Even though the profile of EpFAs were studied at 30 min post ic.v. dosing, a small pilot experiment was performed by testing PTZ 10 min after ic.v. administration. In this assay the regioisomeric mixture of EET methyl esters as well as the sEHI were efficacious in delaying the onset of seizures (Kruskal-Wallis One Way ANOVA on Ranks followed by Dunn's multiple comparison, clonus *p≤0.05, tonus #p≤0.05). (TIF) [file pone.0080922.s003.tif]

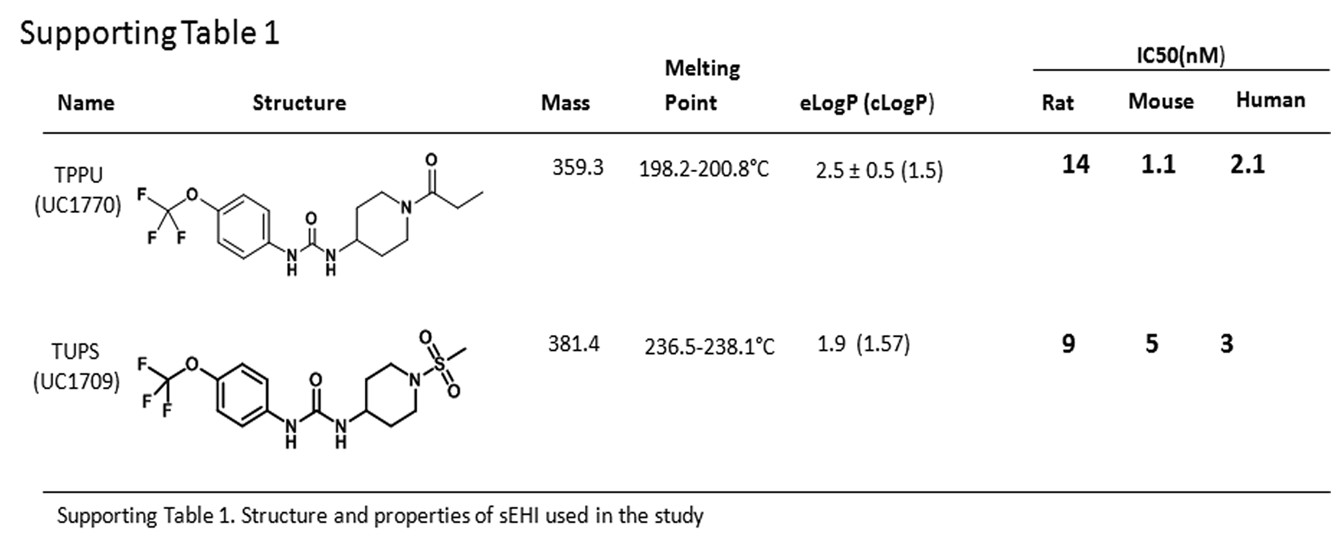

Supplement: Table S1 — Structure and properties of sEHI used in the study. (TIF) [file pone.0080922.s004.tif]
